# Selection and characterization of a DNA aptamer inhibiting coagulation factor XIa

**DOI:** 10.1038/s41598-017-02055-x

**Published:** 2017-05-18

**Authors:** David A. Donkor, Varsha Bhakta, Louise J. Eltringham-Smith, Alan R. Stafford, Jeffrey I. Weitz, William P. Sheffield

**Affiliations:** 1 Centre for Innovation, Canadian Blood Services, Hamilton, Ontario, Canada; 2 Department of Pathology and Molecular Medicine, McMaster University, Hamilton, Ontario, Canada; 3 Departments of Medicine and Biochemistry and Biomedical Sciences, McMaster University and the Thrombosis and Atherosclerosis Research Institute, Hamilton, Ontario, Canada

## Abstract

Factor XIa (FXIa) is a serine protease that catalyzes the activation of Factor IX (FIX) in the blood coagulation cascade. FXIa and its precursor FXI are emergent therapeutic targets for the development of safer anticoagulant agents. Here, we sought a novel DNA-based agent to inhibit FXIa. Towards this goal, an 80 base, single-stranded DNA aptamer library (containing a 40 base randomized core) was screened for FXIa-binding candidates, using ten rounds of positive and negative selection. After selection, 6 of 89 different sequences inhibited FXIa-mediated chromogenic substrate S2366 cleavage. The most active anti-FXIa aptamer had a hypervariable central sequence 5′-AACCTATCGGACTATTGTTAGTGATTTTTATAGTGT-3′ and was designated Factor ELeven Inhibitory APtamer (FELIAP). FELIAP, but not a scrambled aptamer control (SCRAPT), competitively inhibited FXIa-catalyzed S2366 cleavage, FIX activation, and complex formation with antithrombin. No effect of FELIAP on FXI activation was observed. FELIAP inhibited plasma clotting and thrombin generation assays to a significantly greater extent than SCRAPT. Immobilized FELIAP bound FXIa with strong affinity and an equilibrium binding constant (K_D_) in the low nanomolar range determined using surface plasmon resonance. FELIAP is the first FXIa-inhibitory aptamer to be described and constitutes a lead compound to develop related aptamers for *in vivo* use.

## Introduction

The coagulation system can function in a protective or pathological manner. Haemostatic blood clots prevent excessive blood loss at sites of vascular injury^1^, whereas thrombotic clots occlude blood vessels and prevent the flow of blood to critical organs, such as the heart or brain^2, 3^. Thrombosis is responsible for one in four deaths worldwide^4^. Therefore, there is a need for effective and safe anticoagulants to prevent and treat thrombotic disorders.

Currently available anticoagulants include vitamin K antagonists, such as warfarin, and direct oral anticoagulants; dabigatran, rivaroxaban, apixaban and edoxaban. Warfarin attenuates clotting by reducing the hepatic synthesis of multiple coagulation factors^5^, whereas dabigatran inhibits thrombin and rivaroxaban, apixaban and edoxaban inhibit activated factor X (FXa)^6^. The direct oral anticoagulants are at least as effective as warfarin, but produce less bleeding, particularly less intracranial bleeding^6^. Nonetheless, serious bleeding can occur even with the direct oral anticoagulants^7^. Therefore, the search for safer anticoagulants continues.

FXI has emerged as a promising target for safer anticoagulants^8, 9^. FXI is a 160 kDa homodimer comprising two identical disulphide-linked polypeptide chains; specific proteolysis of the Arg^369^-Ile^370^ bond, mediated either by FXIIa or thrombin, converts FXI from an inactive precursor to enzymatically active FXIa^10^. FXIa catalyzes the conversion of FIX to FIXa^10^, which leads to FXa and thrombin generation. Basic and epidemiological studies indicate that FXI is important in thrombosis^11–16^. In contrast, FXI has little role in hemostasis because patients with congenital FXI deficiency rarely have spontaneous bleeding and only bleed with surgery or trauma^17^. Consequently, inhibition of FXI has the potential to attenuate thrombosis without impairing hemostasis. In support of this concept, knockdown of FXI in patients undergoing elective knee replacement was more effective than enoxaparin, the current standard of care, at preventing postoperative venous thromboembolism and did not increase the risk of bleeding^18^. Therefore, there is a push for development of FXI inhibitors.

DNA and RNA ligands, or aptamers, are short single-stranded oligonucleotides (ssDNA or ssRNA) that can be isolated from complex combinatorial libraries of nucleic acids using an iterative *in vitro* selection procedure called systematic evolution of ligands by exponential enrichment (SELEX)^19^. SELEX selects for ssDNA or ssRNA molecules able to adopt stable three-dimensional structures and bind molecular targets from a pool of ~10^14^ unique strands^20^. Although aptamers against numerous coagulation factors have been developed, to our knowledge none have targeted FXIa^21–27^. Here, we describe the selection and characterization of a DNA aptamer that binds the active site of FXIa and inhibits its enzymatic action on both artificial and natural substrates.

## Results

### Selection of FXIa-binding aptamer from a combinatorial library

**Figure 1:**
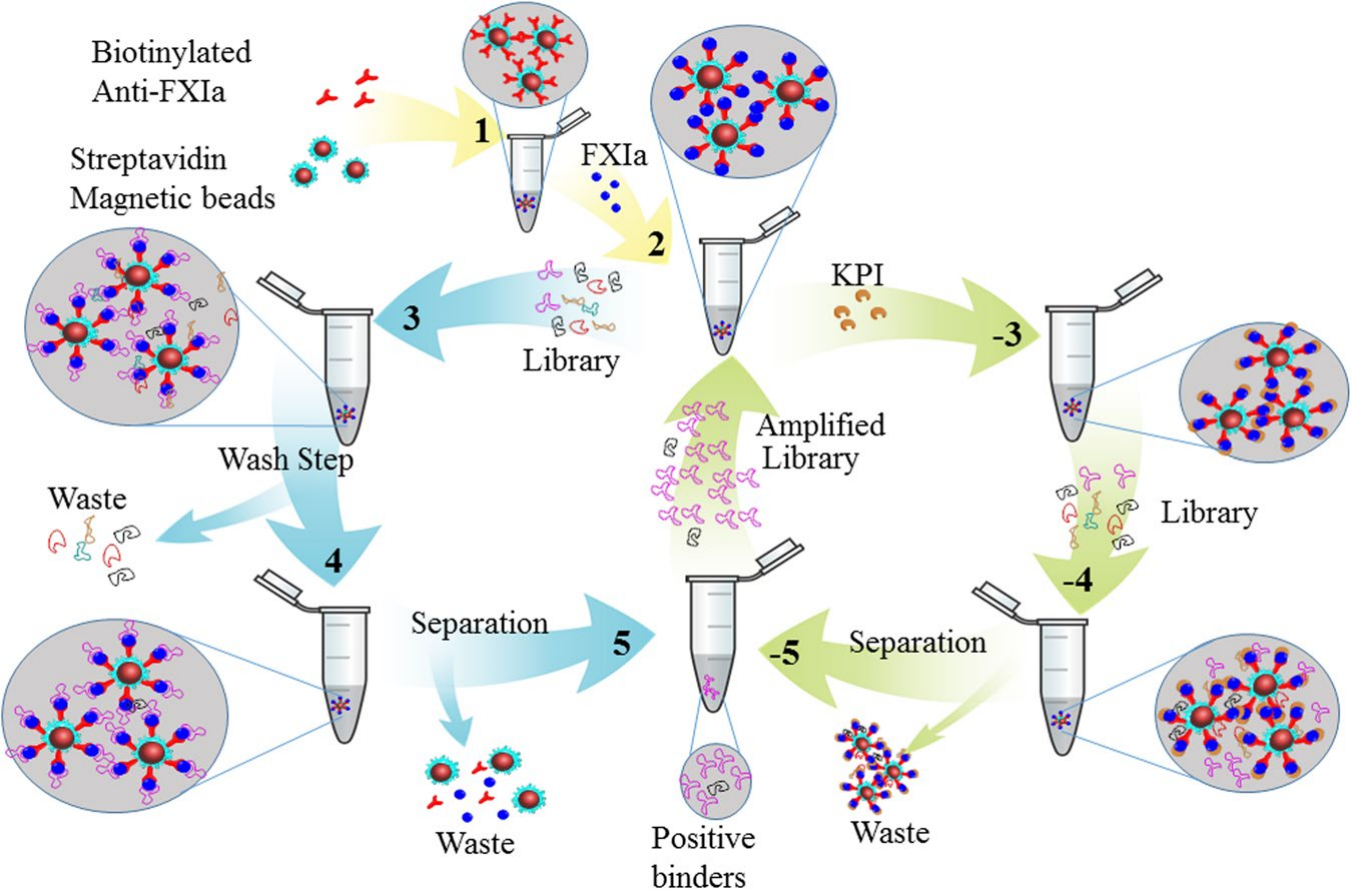
Schematic representation of aptamer library screening strategy. For rounds in which only positive selection was employed, biotinylated anti-FXIa antibodies and streptavidin-coated magnetic beads (1) were combined with FXIa (2) and the original aptamer library (3). Aptamer-FXIa-antibody-bead complexes were then concentrated magnetically and washed (4) prior to separation and recovery of selected aptamers via phenol/chloroform/isoamyl alcohol extraction and ethanol precipitation (5). Following asymmetric PCR to amplify the aptamer pool, steps 1–5 were either repeated directly or components from 1 and 2 were combined with the FXIa-active site inhibitor KPI (−3) for negative selection, magnetic concentration and washing (−4), separation (−5) and amplification, to complete rounds in which both positive and negative selection were employed.

Our objective was to select FXIa-inhibiting aptamers from a large library of ssDNA molecules 80 nucleotides in length containing an internal randomized 40 nucleotide region flanked by primer binding sites. Such a library theoretically contains 4^40^ different DNA molecules. As shown in Fig. 1, an *in vitro* aptamer selection protocol was employed. Initially, we employed only positive selection to enrich for aptamers binding to FXIa. After 4 and 10 rounds of selection, we noted no inhibition of FXIa-mediated amidolysis when the selected aptamer pool was introduced into the reaction (data not shown). Accordingly, we modified the selection protocol by the addition of alternating positive and negative selection steps and rescreened the initial library. The modified protocol included negative selection of aptamers binding to any component of the FXIa-antibody-bead assemblies except the FXIa active site, by introducing the FXIa active site-binding, small protein inhibitor KPI^28^, after Round 4. In contrast to our initial results, after Round 10, a small but reproducible reduction in amidolysis was observed in the presence of the selected aptamer pool.

Sequencing of the pool after Round 4 indicated that the number of unique sequences had been reduced to 289; Table 1 shows the ten most abundant of these sequences. None elicited any inhibition of FXIa activity when tested individually. The majority of these abundant aptamer sequences contained variable sequences of 21 to 23 nucleotides, rather than the 40 nucleotide variable sequences present in the initial library. By Round 10, the selected pool contained only 79 different sequences, as judged by high-throughput sequencing; eight of the ten most abundant sequences were 36–40 nucleotides long. Two abundant sequences, Apt10-1 and Apt10-3, were also found in the Round 4 pool (Apt4-1 and Apt4-2). When the ten most abundant sequences in the Round 10 pool were tested individually, a single aptamer, Apt10-10, was found to inhibit FXIa-mediated cleavage of chromogenic substrate S2366. This tenth most abundant aptamer sequence in Round 10 was designated Factor ELeven(a) Inhibitory Aptamer (FELIAP). When the initial screen of the aptamer library that involved only positive selection with FXIa was continued in parallel to the positive/negative approach, both anti-FXIa activity of the pool and the presence of FELIAP in the pool were noted at Round 20 (data not shown).

**Table 1:** High-throughput sequencing and activity screen data.

Round	N_40_ Variable Sequence	Name (length in nucleotides)	FXIa Inhibition
4	GCGTCCAACACATCGTATTCAT	Apt4-1 (22)	−
4	TGGGATGGCGTGGGAGGGCTGTAGGGAGCGTTCAGTGGGT	Apt4-2 (40)	NT
4	GGGAGGGCGTGGATGGCTGGTGTGAGGTCTTGTGTTTGTT	Apt4-3 (40)	−
4	GGGAGCGTTCAGTGGGT	Apt4-4 (17)	NT
4	GCGTCCAACACATCGGATGATAT	Apt4-5 (23)	NT
4	TGCGTCCAACACATCGTATTCAT	Apt4-6 (23)	NT
4	TGGGATGGCGTGGGAGGGCTGTAGTGAGCGTTCAGTGGGT	Apt4-7 (40)	NT
4	CGTCCAACACATCGTATTCAT	Apt4-8 (21)	NT
4	CTTGCCCACTATCGACTTCACC	Apt4-9 (22)	NT
4	GCGTCCAACACATCGTAAGTA	Apt4-10 (21)	NT
10	GCGTCCAACACATCGTATTCAT	Apt10-1 (22)	−
10	CACTGCGTCCAACACATCGTATTCAT	Apt10-2 (26)	−
10	TGGGATGGCGTGGGAGGGCTGTAGGGAGCGTTCAGTGGGT	Apt10-3 (40)	−
10	TGGGATGGCGTGGGAGGGCTGTAGTGAGCGTTCAGTGGGT	Apt10-4 (40)	−
10	GGGAGGGCGTGGATGGCTGTTGTGAGGTCTTGTGTTTGTT	Apt10-5 (40)	−
10	TGGGATGGCGTGGGAGGGCTGTAGGGAGCGTTTAGTGGGT	Apt10-6 (40)	−
10	TGGGATGGCGTGGGAGGGCTGTAGTGAGCGTTCATTGGGT	Apt10-7 (40)	−
10	GGGAGGGCGTGGATGGCTGGTGTGAGGTCTTGTGTTTGTT	Apt10-8 (40)	−
10	TGGGATGGCGTGGGAGGGCTGTAGTGAGCGTTTAGTGGGT	Apt10-9 (40)	−
10	AACCTATCGGACTATTGTTAGTGATTTTTATAGTGT	Apt10-10 (36)	+

DNA sequence of aptamers (name and length in nucleotides, column 3) isolated after 4 or 10 rounds of screening (column 1) corresponding to the N_40_ variable domain in the original aptamer library (5′-GAATTCTAAT ACGACTCACT ATA-N_40_-GCGTCCAACA CATCG-3′) is given in column 2. Whether (+) or not (−) the aptamer had inhibitory activity when introduced into FXIa-mediated amidolysis of S2366 is shown in column 4. The ten most abundant sequences obtained after Rounds 4 or 10 are shown in rank order; NT signifies not tested.

### Comparison of FELIAP to related aptamer candidates

**Figure 2:**
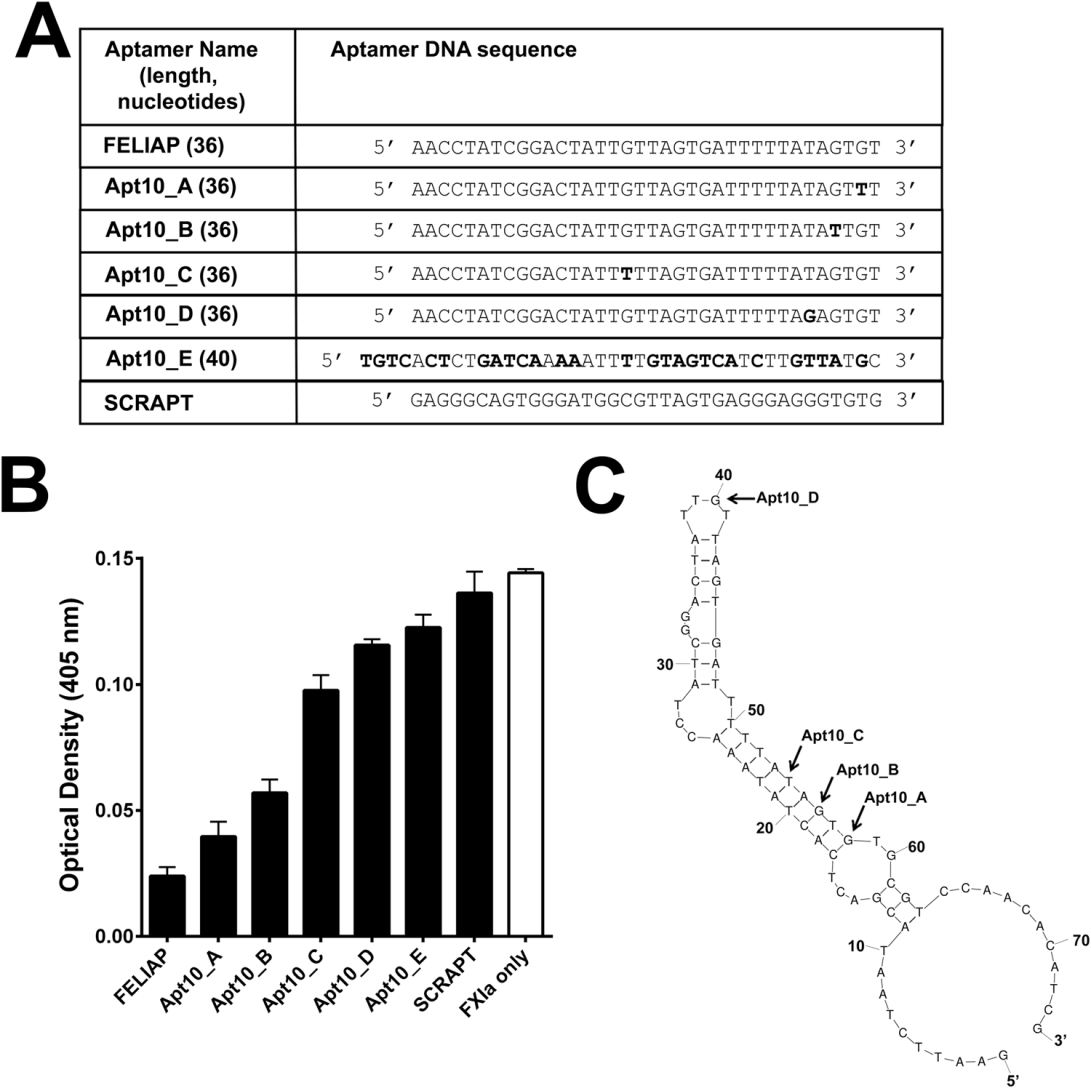
Characterization of Round 10 aptamers as inhibitors of FXIa-mediated amidolysis. (**A**) Means ± SEM (n = 3) of colour generation following FXIa-mediated amidolysis of chromogenic substrate S2366, in the presence (black bars) of Round 10 aptamers identified on the x axis or a scrambled negative control sequence (SCRAPT), or in the absence of added DNA molecules (white bar). (**B**) Mfold-generated predicted secondary structure of FELIAP. The positions of base substitutions between FELIAP and other Round 10-selected aptamers are indicated by arrows. (**C**) DNA sequence of Round 10 aptamers and SCRAPT. The sequence corresponding to the original 40 nucleotide hypervariable core of the aptamer library is shown, with base substitutions relative to FELIAP in bold.

Comparison of FELIAP to the other 78 sequences of lesser abundance found in Round 10 revealed five related aptamers, four of which (Apt10_A through D) were closely related, differing in at most two of 36 positions; the fifth (Apt10_E) was only distantly related by virtue of T-rich areas (38.9% identical). The aligned sequences are shown in Fig. 2A, as well as that of a scrambled aptamer control (SCRAPT). When these aptamers were tested for their ability to inhibit FXIa amidolysis, FELIAP was found to be the most active and Apt10_E the least active, although all six selected aptamers showed greater inhibitory activity than SCRAPT (Fig. 2B). Modeling of predicted secondary structure using the Mfold web server^29^ showed that the portion of FELIAP corresponding to the variable part of the aptamer library likely adopted an extended stem-loop structure containing a bulge separated by two small loops (Fig. 2C). Three of four substitutions correlating with reduced anti-FXIa activity were found in a predicted extended 9 base pair stem structure at the distal end of this structure, while the substitution in APT10_C was at the very apex of the predicted hairpin. As FELIAP was the most potent inhibitor identified in the library, it was employed in all subsequent experiments.

### Kinetic characterization of FELIAP as an inhibitor of FXIa-mediated amidolysis and clotting

**Figure 3:**
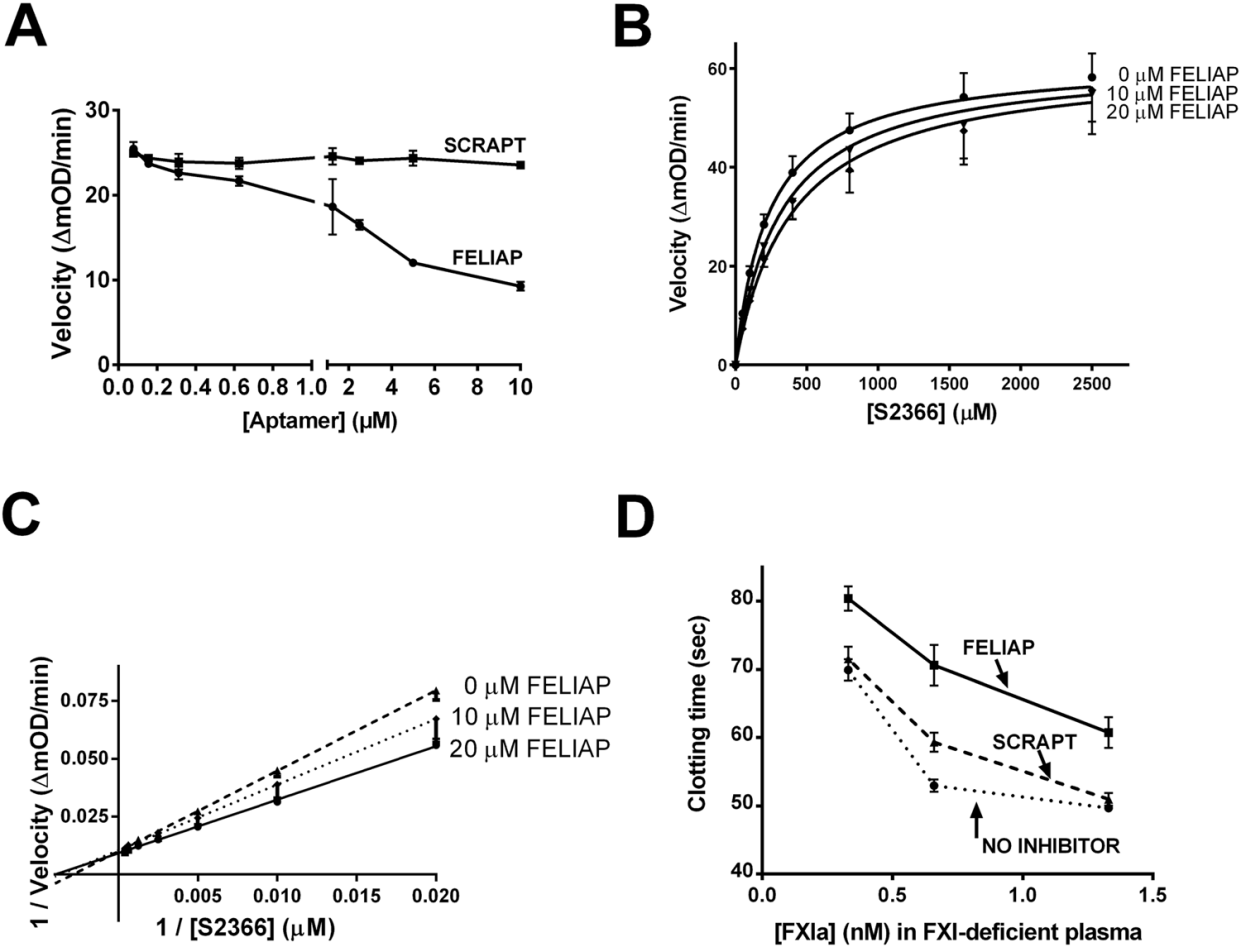
Kinetic characterization of FELIAP inhibition of FXIa-mediated amidolysis. (**A**) Means ± SEM (n = 3) of reaction velocity for FXIa-mediated amidolysis of S2366 versus aptamer concentration (FELIAP, circles; SCRAPT, squares). (**B**) Means ± SEM (n = 3) of reaction velocity versus S2366 concentration in the presence of 0, 10, or 20 µM FELIAP. (**C**) Lineweaver-Burke transformation of data in (**B**). (**D**) Means ± SEM (n = 7) of modified APTT assays in which FELIAP or SCRAPT were pre-incubated with FXIa, at concentrations given on the x axis, prior to dilution into recalcified FXI-deficient plasma.

The mode of inhibition of FELIAP was investigated by fixing the concentrations of chromogenic substrate and FXIa and increasing the concentration of FELIAP and measuring the rate of substrate cleavage. A dose-dependent reduction in the reaction rate was observed as FELIAP was increased from 0 to 20 µM, while the same concentrations of SCRAPT had no inhibitory effect (Fig. 3A). Next, the concentration of chromogenic substrate S2366 was varied while keeping FXIa constant at different concentrations of FELIAP (Fig. 3B). The reaction demonstrated competitive inhibition, as suggested by the apparent lack of alteration of the maximum reaction velocity (Fig. 3B) with increasing FELIAP concentrations, and by the common y intercept on the Lineweaver-Burke transformation of the velocity versus substrate curves for increasing FELIAP concentrations (Fig. 3C). Fitting the curves to a competitive inhibition model yielded an estimated K_i_ for FELIAP of 29 µM by non-linear regression.

Prior to examining the inhibitory effects of FELIAP on FXIa-mediated interactions with specific macromolecular substrates, we examined the capacity of FELIAP to inhibit FXIa-induced clotting in plasma. FXIa was pre-incubated with buffer, FELIAP, or SCRAPT, and combined with FXI-deficient plasma and APTT reagent containing kaolin and cephalin, and recalcified. As shown in Fig. 3D, at all three FXIa concentrations tested, FELIAP delayed plasma clot formation to a greater extent than SCRAPT or buffer controls.

### Effects of FELIAP on macromolecular reactions of FXI and FXIa

**Figure 4:**
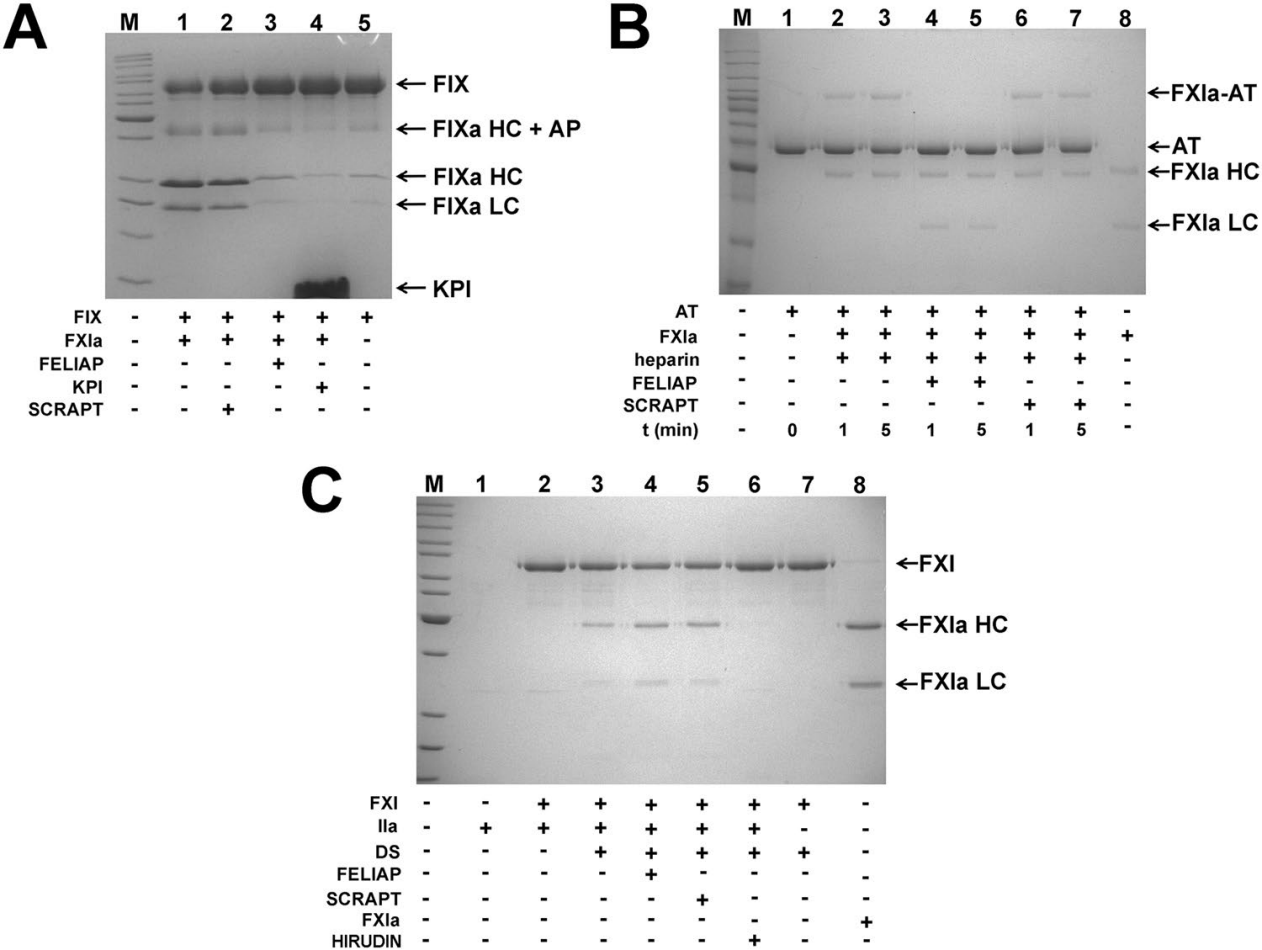
Effects of FELIAP on reactions of FXIa and FXI with macromolecular substrates. (**A**) Coomassie-stained reduced SDS-polyacrylamide gel. FIX was reacted with FXIa in the presence (+) or absence (−) of SCRAPT (lane 2), FELIAP (lane 3) or KPI (lane 4). Arrows and labels, at right, identify the position of FIX, forms of FIXa (including HC, heavy chain, HC + AP, heavy chain + activation peptide intermediate, LC, light chain) and KPI. M, markers (kDa), at left: 220; 160; 120; 100; 90; 80; 70; 60; 50; 40; 30; 25; 20; 15. (**B**) As in A, except antithrombin (AT, lane 1) was reacted with FXIa (lane 8) in the presence of heparin for 1 (lane 2) or 5 (lane 3) minutes with FELIAP (lanes 4 and 5) or SCRAPT (lanes 6 and 7) or no DNA addition (lanes 2 and 3). Arrows and labels identify the position of AT, FXIa-AT covalent complex, FXIa heavy chain (HC) and light chain (LC). M, markers (kDa), at left: 220; 160; 120; 100; 90; 80; 70; 60; 50; 40; 30; 25; 20 (**C**). As in **A** and **B** except FXI and thrombin (IIa, lane 1) were reacted in the absence (lane 2) or presence of dextran sulphate (DS, lanes 3–7) with the addition of FELIAP (lane 4) or SCRAPT (lane 5) or the thrombin inhibitor hirudin (lane 7). Lane 8, purified FXIa. M, markers (kDa), at left: 220; 160; 120; 100; 90; 80; 70; 60; 50; 40; 30; 25; 20; 15.

FXIa activates FIX by cleaving two peptide bonds, one at Arg^145^-Ala^146^, and the other at Arg^180^-Val^181^ of the FIX polypeptide^30^. This reaction liberates a glycosylated activation peptide (Ala^146^-Arg^180^, 10 kDa), and a disulphide-linked γ-carboxylated light chain (Tyr^1^-Arg^145^, 25 kDa) and heavy chain (Val^181^-Thr^405^, 30 kDa)^31, 32^. An intermediate product comprised of the activation peptide linked to the heavy chain (Ala^146^-Thr^405^, 40 kDa) may also be detected^31^. Figure 4A shows that FXIa-mediated FIX activation was unaffected by SCRAPT (compare heavy and light chains, lanes 1 and 2), while introduction of excess FELIAP (lane 3) or KPI (lane 4) reduced FIXa generation to background levels (lane 5, no FXIa added).

FXIa also reacts with its natural inhibitor, antithrombin, in a reaction that is accelerated by heparin, to form a denaturation-resistant complex between the FXIa active site and the reactive centre of antithrombin, in which the light chain of FXIa (Ile^370^-Val^607^) is joined to antithrombin residues His^1^-Arg^393^ via an acyl linkage^10, 33^. Figure 4B shows SDS-PAGE evidence of this 90 kDa complex after 1 or 5 minutes of reaction (lanes 2 and 3) and in the presence of excess SCRAPT (lanes 6 and 7) but not in the presence of excess FELIAP (lanes 4 and 5).

Having demonstrated that FELIAP inhibited the action of FXIa on two macromolecular substrates, FIX and antithrombin, we next asked if it had any effect on the activation of FXI by thrombin. In the presence of cofactor dextran sulphate (Fig. 4C, lane 3), but not its absence (lane 2), FXI was efficiently activated into FXIa, as previously reported^34^. No inhibition of this reaction was noted in the presence of either excess FELIAP (lane 4) or SCRAPT (lane 5), but activation of FXI was abrogated in the presence of excess hirudin, a potent thrombin inhibitor (lane 6). FELIAP therefore inhibited FXIa-dependent but not FXI-dependent reactions.

### Inhibition of thrombin generation in plasma by FELIAP

**Figure 5:**
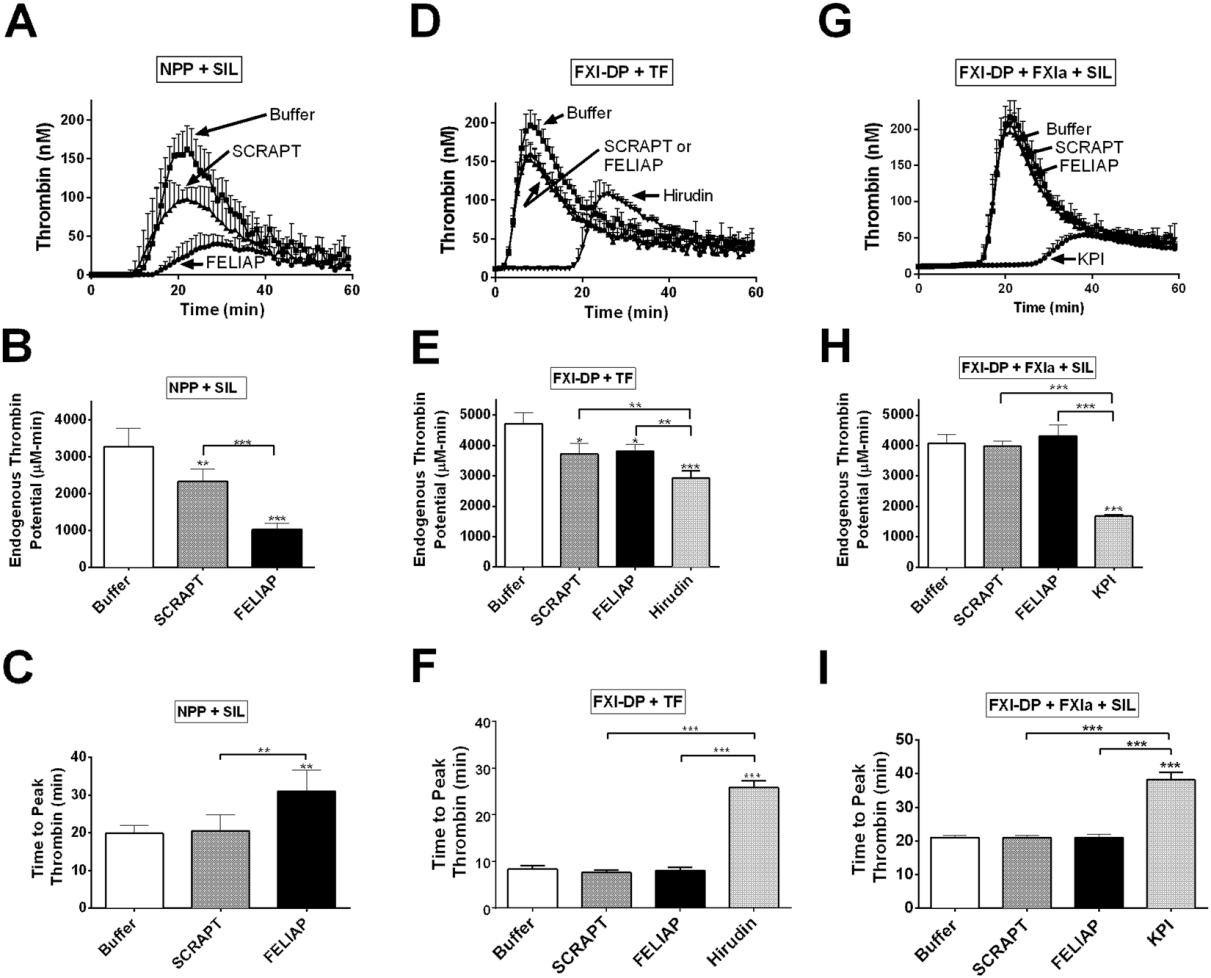
Inhibition of thrombin generation by FELIAP. Thrombin generation assays (TGA) were conducted in 3 different ways: in recalcified human normal pooled plasma (NPP) using micronized silica (SIL) for contact activation as the initiator (NPP + SIL, panels A-C); in FXI-depleted plasma (FXI-DP) using tissue factor for extrinsic pathway activation as the initiator (FXI-DP + TF, panels D-F); and following 0.25 nM FXIa pre-incubation with Buffer, 1 µM SCRAPT, or 1 µM FELIAP, in FXI-DP activated with micronized silica (FXI-DP + FXIa + SIL). Thrombin concentration was determined fluorescently every minute for 60 minutes in each case. (**A**) TGA progress curves (mean ± SD (n = 6) with addition of agents (Buffer, 30 μM FELIAP, or 30 μM SCRAPT) as indicated by labels and arrows. Upwards error bars are shown. (**D**) As in A, but n = 5, and with additional assays including 0.2 µM recombinant hirudin variant 3 (Hirudin). (**G**) FELIAP, SCRAPT or KPI was pre-incubated with FXIa (1 µM: 0.25 mM inhibitor: enzyme ratio) and then diluted into FXI-DP activated with micronized silica for TGA. Progress curves (mean ± SD (n = 5)) are shown. Each set of TGA progress curves (**A**,**D**,**G**) are quantified with respect to endogenous thrombin potential (the area under the thrombogram curve) (**B**,**E**,**H**) and time to peak thrombin (**C**,**F**,**I**) in panel columns. Bar graphs are derived from analysis of individual thrombogram curves corresponding to plasma supplementation with Buffer (white), SCRAPT (grey), FELIAP (black), or, in some reactions KPI or Hirudin (light grey). Symbols (above the error bars) indicate statistically significant differences from Buffer reactions, while symbols above the horizontal bar indicate statistically significant differences between SCRAPT- and FELIAP-supplemented and other reactions: p < 0.001, *; *p < 0.001, ***.

Recalcified dilute normal human pooled plasma, in which the contact pathway of coagulation was activated by micronized silica, was employed to assess the effects of FELIAP on thrombin generation. The thrombin generation assay (TGA) provides information on the timing and kinetics of thrombin generation in plasma using a thrombin-specific fluorogenic substrate and calibrators unaffected by fibrin clot formation, using standardized analytic methods and parameters^35^. As shown in Fig. 5A, FELIAP had greater effects on thrombin generation in recalcified dilute plasma than SCRAPT. Firstly, 30 µM FELIAP prolonged the lag time of thrombin generation relative to either 30 µM SCRAPT or buffer controls (data not shown). Similarly, FELIAP reduced the endogenous thrombin potential (ETP; the area under the thrombin generation curve) by 3.2-fold, versus 1.4-fold for SCRAPT (Fig. 5B; both reductions p < 0.001). Finally, the mean time to peak thrombin was increased 1.5-fold by FELIAP but was unaffected by SCRAPT (Fig. 5C).

The partial anticoagulant activity observed for SCRAPT in silica-activated TGA using normal pooled plasma was also noted for other single-stranded oligonucleotides of similar length (70–80 nucleotides; data not shown) at equimolar concentrations. To ascertain whether or not this phenomenon was related to FXIa inhibition, we repeated TGA, substituting FXI-depleted plasma for normal plasma and tissue factor for silicates as activators (Fig. 5D). SCRAPT or FELIAP exhibited indistinguishable 1.24- to 1.27-fold reductions in endogenous thrombin potential relative to buffer under these conditions (Fig. 5E), but unaltered times to peak thrombin (Fig. 5F). In contrast, the specific thrombin inhibitor hirudin, at 200 nM, elicited a significantly greater reduction in ETP than 30 µM FELIAP or SCRAPT, and extended the time to peak thrombin by 3.1-fold (p < 0.001 versus FELIAP, SCRAPT or buffer, Fig. 5F). SCRAPT or FELIAP effects were eliminated when the aptamers (1.0 µM) were combined with 0.25 nM FXIa and then combined with FXI-depleted plasma activated by silicates (Fig. 5G–I). In contrast, substitution of KPI for either aptamer significantly reduced the ETP and the time to peak thrombin (Fig. 5G–I). FELIAP therefore inhibited TGA to a greater extent than SCRAPT in plasma activated via the contact pathway, but not the extrinsic pathway, but to a considerably lesser extent than KPI.

### Use of Surface Plasmon Resonance (SPR) to characterize FELIAP binding to FXIa

**Figure 6:**
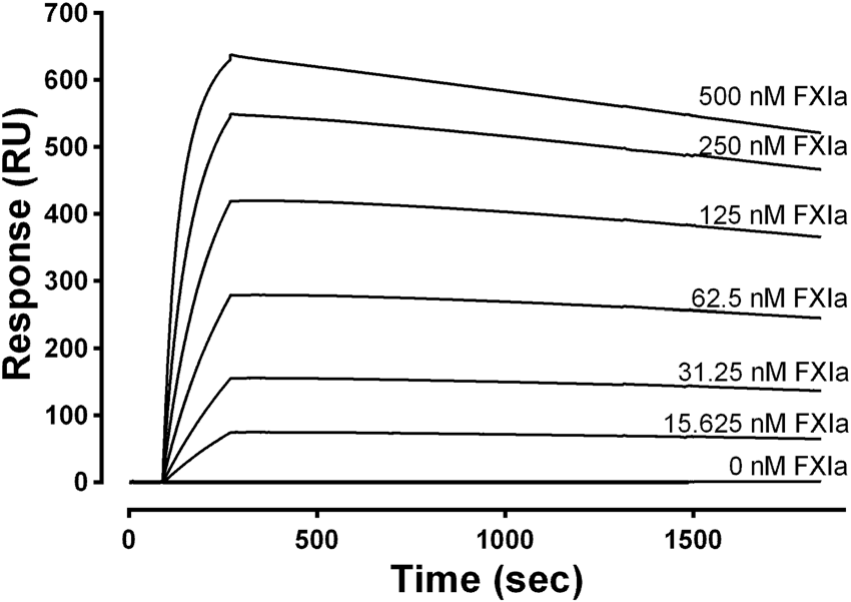
Binding of FELIAP to immobilized FXIa. An SPR sensogram showing the interactions between 3′biotinylated FELIAP immobilized on a streptavidin-coated chip and FXIa at 25 °C at FXIa concentrations given above each progress curves. Association was allowed to proceed for 180 seconds followed by 1800 seconds of dissociation. All curves were corrected for non-specific effects by signal subtraction using a reference cell containing 3′biotinylated SCRAPT immobilized on a streptavidin-coated chip. A single dilution series of curves representative of a total of 3 others is shown.

FELIAP and SCRAPT were biotinylated at their 3′ ends and immobilized on a streptavidin-coated gold chip. Immobilized SCRAPT served as the reference cell. When increasing concentrations of FXIa from 0 to 500 nM were flowed over these surfaces, increasing response unit binding isotherms were generated when the difference between immobilized FELIAP and immobilized SCRAPT binding was plotted (Fig. 6). It should be noted that the maximum response for SCRAPT binding did not exceed 25 response units at 500 nM FXIa, 1800 seconds (data not shown). The net binding isotherm was characterized by relatively rapid association kinetics and very slow dissociation kinetics. Analysis of these isotherms yielded values for dissociation rate constant (K_d_) and the association rate constant (K_a_), permitting calculation of the equilibrium binding constant K_D_, which is K_d_ divided by K_a_. K_a_ values of 5.2 ± 0.1 × 10^4^ M^−1^ S^−1^, K_d_ values of 9.5 ± 0.1 × 10^−5^ S^−1^, and K_D_ values of 1.8 ± 0.1 × 10^−9^ M were obtained (mean ± SD of three determinations).

### Effects of progressive truncation of FELIAP on inhibition of FXIa-mediated amidolysis

**Figure 7:**
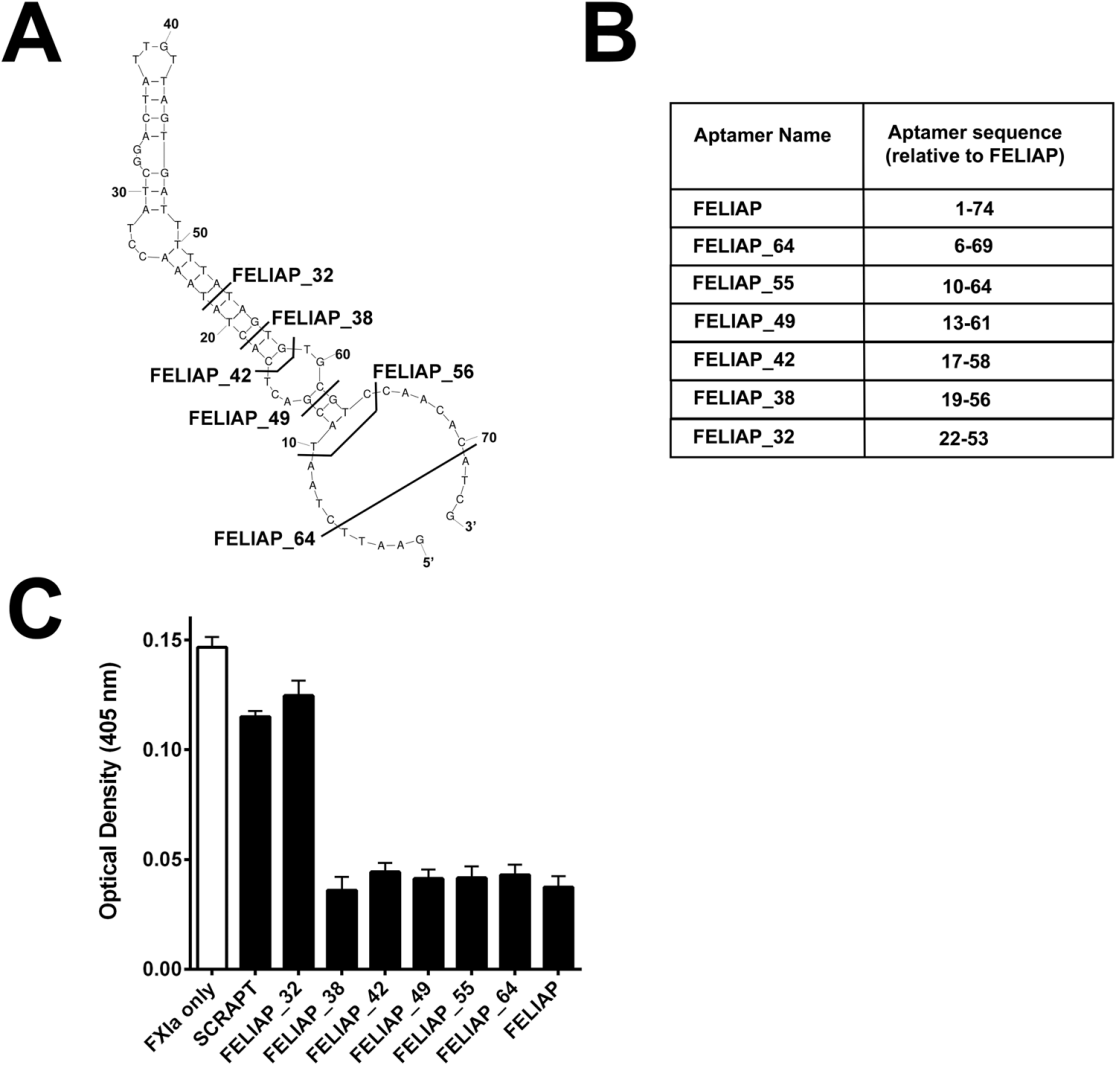
Characterization of truncated derivatives of FELIAP as inhibitors of FXIa-mediated amidolysis. (**A**) Extent of truncation analysis. Sequences to the left of bolded lines crossing the Mfold-generated predicted secondary structure of FELIAP are identified as FELIAP_X, where X = the length of the truncated aptamer (32, 38, 42, 49, 55, 64, or 74 for full-length FELIAP. (**B**) Tabular depiction of truncated aptamers relative to FELIAP; for instance, FELIAP_32 (relative sequence 22–53) is identical to FELIAP except for the deletion of 21 nucleotides from the 5′end and 21 nucleotides from the 3′ end of FELIAP. (**C**) Means ± SEM (n = 3) of colour generation following FXIa-mediated amidolysis of chromogenic substrate S2366, in the presence (black bars) of SCRAPT, FELIAP, or truncated derivatives of FELIAP identified in panels A and B, or in the absence of of added DNA molecules (white bar).

To determine the minimum active sequence of FELIAP required for inhibition of FXIa, several truncated forms of FELIAP were synthesized, using the predicted structure of the aptamer to delete predicted loops and stems progressively. The truncated sequences ranged from 32 to 64 nucleotides long (compared to 74 for full-length FELIAP), were designated FELIAP_X (where X = 32, 38, 49, 55 and 64), and constituted 5′ and 3′ deletion mutants of FELIAP (see Fig. 7A and B). As shown in Fig. 7C, deletion of two predicted loop structures and an intervening short stem (in FELIAP_38) had no effect on anti-FXIa activity. However, reduction of the predicted terminal stem structure from seven (in FELIAP_38) to four base pairs (in FELIAP_32) reduced anti-FXIa activity to background levels equivalent to SCRAPT.

## Discussion

Aptamers from combinatorial libraries can be selected to bind virtually any protein of interest. Here, we describe the selection and characterization of FELIAP, a FXIa-binding DNA aptamer. Our protocol favoured the selection of aptamers binding at or near the active site of FXIa over those binding to other portions of the enzyme. Several lines of indirect evidence support the conclusion that this strategy was successful. Firstly, FELIAP acted as a competitive inhibitor of chromogenic substrate S2366, which is a tripeptide nitroanilide compound (pyroglutamyl-prolyl-arginyl-*p*-nitroanilide) which must, by virtue of its small size, enter the interior of the FXIa active site pocket to be cleaved and liberate the coloured product nitroanilide. Secondly, it inhibited two different FXIa-dependent reactions: FXIa-mediated activation of FIX; and FXIa-mediated formation of FXIa-antithrombin complexes. While this observation does not *per se* exclude an allosteric effect of FELIAP on FXIa, it renders it less likely than active site binding. Thirdly, FELIAP had no effect on FXI activation, further indicating specificity for FXIa, one of whose cardinal features is the active site. Taken together, these data suggest that FELIAP binds specifically to FXIa at or near its active site, with high affinity consistent with the observed nanomolar K_D_.

We employed KPI to obscure the active site of FXIa in KPI-FXIa-anti-FXI-bead assemblies for negative selection, reasoning that depletion of the aptamer library of candidates binding to any of the constituent parts of these assemblies would enrich for those binding the FXIa active site. KPI is the Kunitz protease inhibitor domain of protease nexin 2, which is an isoform of the β-amyloid precursor protein (APP) secreted from α-granules on platelet activation. The 57 amino acid KPI domain accounts for all of the FXIa-inhibitory activity of APP^36^ and has been crystallized in complex with FXIa^28^. The crystal structure revealed that two loops in KPI formed extensive contacts with FXIa; in particular residues in the KPI Thr^11^-Arg^20^ loop extend into the substrate pocket, rationalizing the high affinity binding evidenced by reported K_i_ values of 300–500 pM. While it may seem somewhat circular to use a polypeptide active site inhibitor to identify a ssDNA aptamer inhibitor, aptamers provide multiple potential advantages over proteinaceous inhibitors with respect to immunogenicity, cost of production, antidote generation, and the potential to fine-tune binding via mutagenesis^37^.

While to our knowledge no aptamers to FXI or FXIa have been previously described in the biomedical literature, several aptamers to other coagulation factor targets have been isolated.

Among RNA aptamers, RNA 16.3 bound FVII or FVIIa with K_D_ values of 10–13 nM, inhibiting the enzyme by disrupting tissue factor-FVII(a) complex assembly^22^. A truncated form of a selected RNA aptamer designated 9.3t was found to bind FIXa with a K_D_ of 0.58 nM^38^, specifically at an extended substrate binding position, or exosite^39^. RNA_11F7t_ aptamer bound FXa with a reported K_D_ of 1.1 nM and inhibited prothrombinase activity by interfering with the interaction between FXa and FVa^21^, while R4CxII-It bound FXII or FXIIa with K_D_ values of 8.9 and 0.4 nM, respectively, by interfering with FXII and anionic binding^24^. RNA_R9D-14T_ bound thrombin or prothrombin with K_D_ values of 1 or 10 nM via anion binding exosite I^23^. With respect to DNA aptamers, the only ones described to date that target coagulation factors are HD1 and HD22, which bind prothrombin with K_D_ values of 7.1 and 2.4 nM, via exosites I and II, respectively^40, 41^. All previously described aptamers targeting coagulation factors, therefore, act at sites distinct from the active site of these serine proteases. Our discovery of FELIAP, which acts at or near the active site of FXIa, may have been facilitated by the use of KPI in negative selection, as screening protocols employed in previously published studies used only positive selection. Although the time- and labour-intensive nature of aptamer library screening precluded a systematic analysis, anecdotally we did observe FELIAP selection within ten rounds of screening with positive and negative steps, but not until twenty rounds of screening with positive steps alone.

Although SELEX and SELEX-related aptamer screening protocols have been employed to isolate aptamers that bind to their targets with high affinity, numerous limitations need to be overcome for successful application of this biotechnological approach. These include interference from non-degenerate sequences flanking the variable regions in the aptamer library; non-specific retention of sequences not binding the desired target; and accumulation of amplification artifacts and artifactual sequences arising from library regeneration^20^. We observed many examples of such artifacts using high throughput sequencing after Round 4, some of which persisted to Round 10, as well as the selection of aptamers with barely detectable affinity for FXIa (e.g. Apt10_E). Our results are therefore consistent with the known limitations of SELEX.

While FELIAP was found to bind FXIa with high affinity (K_D_ of 1.8 nM), in the general nanomolar range of previously reported aptamers against other coagulation factors, its potency as an inhibitor of FXIa-mediated reactions was less impressive. Its K_i_ for inhibition of FXIa-mediated amidolysis of S2366 was 29 µM, and µM concentrations of FELIAP were required to impair nM concentrations of FXIa with respect to its reactions with FIX or antithrombin or to elicit substantial reduction of thrombin generation in plasma systems. This pattern is consistent with FELIAP binding with high affinity to a location close enough to the active site to yield a competitive inhibition, but in a manner that does not fully block access to the active site for either small molecule or macromolecular substrates. As noted above, an alternative hypothesis would involve high affinity binding to an allosteric site that elicited a partially inhibitory change in the reactive site. FELIAP may therefore be regarded as a lead compound for the development of more potent FXIa-inhibiting aptamers, in the same way as some monosulphated benzofurans have been reported to be lead compounds for the design of small molecule FXIa inhibitors^42^.

Inspection of the Mfold generated secondary structural folding prediction showed that the extended stem-loop structure predicted for FELIAP encompassed more than the variable region nucleotides 24 to 59. FELIAP_38 retained all of full-length FELIAP’s anti-FXIa activity, but only three additional base pairs in a predicted terminal stem than non-inhibitory FELIAP_32. These results, taken together with our finding that mutation of the guanosine residue at the predicted turn of the hairpin at nucleotide 40, support the secondary structure model and likely indicate that the AT-rich stem disrupted in FELIAP_32 must be stabilized by additional A-T hydrogen bonds or by the C-G bonding between residues 19 and 56 in FELIAP_38. It is likely that this stem stabilizes the “loop-bulge-loop” conformation of FELIAP_38 for direct contact with FXIa. Localization of the aptamer within the FXIa active site by cross-linking, modeling or co-crystallization will in future be used to test this working hypothesis and to provide clues to how to increase its potency as an inhibitor to match its high affinity as a FXIa ligand. One way to achieve this goal might be through its derivatization with bulky groups that would further block access to the active site for other substrates. Such optimization will likely be necessary before the FXIa aptamer can be tested in animal models of thrombosis and bleeding; doses of 3.6 mg/kg KPI were required to halve thrombus size in the carotid arteries of mice subjected to ferric chloride injury, and that protein demonstrated a K_i_ four to five orders of magnitude lower for FXIa than FELIAP^43^.

## Materials and Methods

### Reagents

The aptamer library comprised a ssDNA template with sequence 5′-GAATTCTAAT ACGACTCACT ATA-N_40_-GCGTCCAACA CATCG-3′ (please note spaces have been introduced every 10 nucleotides, into this and all other DNA sequences reported here). The forward (A) and reverse primers (B) were 5′-GAATTCTAAT ACGACTCACT ATA-3′ and 5′-GCGTCCAACAC ATCG-3′ respectively. These and all other oligonucleotides employed in this study were purchased from Integrated DNA Technologies (IDT, Coralville, IA). FXI, FXIa, FIX and FXIIa were bought from Enzyme Research Laboratories (South Bend, IN). Biotinylated goat anti-human Factor XI (FXI) antibody was purchased from Affinity Biologicals (Ancaster, ON). Dynabeads Biotin Binder was bought from Thermo Fisher Scientific (Waltham, MA). For thrombin generation assays (TGA), TGA substrate and TGA calibrator sets were obtained from Technothrombin GmbH (Vienna, Austria). Activated Partial Thromboplastin Time (APTT) reagent was from Diagnostica Stago (Asnieres, France). Chromogenic substrate S2366 was purchased from Instrumentation Laboratory (Lexington, MA).

### Systemic Evolution of Ligands by Exponential Enrichment (SELEX)

Solution-based SELEX was performed as previously described^19^ with some modifications, as portrayed schematically in Figure 1. In the sequence of the starting ssDNA library, 5′-GAATTCTAAT ACGACTCACT ATA-N_40_-GCGTCCAACA CATCG-3′, N_40_ represents a 40 nucleotide randomized region. Before selection, streptavidin-coated magnetic beads (5 μL packed volume) were prewashed five times in Aptamer Folding Buffer (AFB; 20 mM Tris-HCl pH 7.4, 140 mM NaCl, 5 mM KCl, 1 mM MgCl_2_ and 1 mM CaCl_2_). All reactions took place at room temperature; washes were performed using a magnet to concentrate the beads at the bottom of the reaction tube. Next, biotinylated goat anti-human factor XI antibody (Affinity Biologicals) was added at quadruple the FXIa concentration to be employed, and incubated for 45 minutes prior to washing. FXIa (60 nM) was then added to the antibody-coated beads and incubated for 1 hour. Five washes were carried out prior to the addition of 1000 picomoles of aptamer library which had been diluted to 4 nM in AFB and heated to 90 °C for 5 minutes before being cooled on ice. The folded library was then added to the immobilized FXIa and incubated for 1 hour with end-over-end rotation on a Barnstead Thermolyne Labquake. Unbound aptamers were removed by five washes in AFB. Bound aptamers were extracted from protein/antibody/bead assemblies using phenol: chloroform: isoamyl alcohol (25:24:1, vol/vol, saturated with Tris-Cl, Thermo Fisher Scientific) and precipitated with 2/3 vol/vol absolute ethanol to conclude the selection round. The pool of selected aptamers was then PCR-amplified using a high-fidelity heat-stable DNA polymerase (Phusion; Thermo Fisher Scientific) in an asymmetric PCR protocol in which 13-fold more primer A than primer B was employed; the ssDNA sense strand was then purified by preparative agarose gel electrophoresis using a 2% (w/vol) agarose gel and an Ultrafree-DA centrifugal filter unit (Millipore Sigma, Billerica, MA) to produce the amplified aptamer library and conclude the first selection round (Round 1). After Round 3, selection stringency was increased by switching from AFB to stringent wash buffer (SWB; 20 mM Tris-HCl pH 7.4, 4 M NaCl, 5 mM KCl, 1 mM MgCl_2_, 1 mM CaCl_2_, 0.005% Tween 20) and gradually reducing the incubation time of the library with FXIa such that it was only 15 min in Round 10.

After five rounds of positive selection, the resulting amplified library was combined with beads, biotinylated anti-FXIa, and FXIa as described above, and negatively selected using recombinant His-tagged Kunitz Protease Inhibitor domain of human protease nexin 2 (KPI, 63 amino acids) expressed in *Pichia pastoris* yeast and purified exactly as described^28^, at a concentration of 6.8 µM, to block the FXIa active site. Washing and incubations were as described above for positive selection, except that aptamers from the amplified library not binding to the KPI-FXIa-antibody-bead assemblies were magnetically separated and combined with fresh FXIa, anti-FXI antibodies and magnetic beads to start the next round of selection. Rounds six through ten of SELEX thus combined positively selecting aptamer candidates with unblocked FXIa and negatively selecting against aptamer candidates binding to KPI-blocked FXIa or anti-FXI antibodies or magnetic beads.

### High-throughput sequencing

High-throughput (also known as deep) sequencing was employed to characterize the selected aptamer populations following four and ten rounds of SELEX. Single-stranded aptamers generated by asymmetric PCR were PCR-amplified using forward primer 5′-AATGATACGGC GACCACCGAG ATCTACACTA GATCGCACAC TCTTTCCCTA CACGACGCTC TTCCGATCTN NNNGAATTCT AATACGACTC ACTATA-3′ and reverse primer 5′-CAAGCAGAAG ACGGCATACG AGATTCGCCT TAGTGACTGG AGTTCAGACG TGTGCTCTTC CGATCTCGAT GTGTTGGACA AGCAGAAGAC GGCATACGAG ATTCGCCTTA GTGACTGGAG TTCAGACGTG TGCTCTTCCG ATCTCGATGT GTTGGACGCC GC-3′. The resulting amplicons were sequenced using an Illumina Miseq DNA sequencer at the Farncombe Metagenomics Facility, McMaster University. The raw sequencing data was processed using Illumina’s Basespace online NGS platform for tagged sequence pool sorting, and to ensure sequence data output was converted to FASTQ format. Further data processing was as described^44^.

### Aptamers

The full length FELIAP aptamer sequence was determined to be 5′-GAATTCTAAT ACGACTCACT ATAAACCTAT CGGACTATTG TTAGTGATTT TTATAGTGTG CGTCCAACAC ATCG-3′. A control aptamer of the same length but scrambled DNA sequence (SCRAPT) was synthesized for comparative purposes, with sequence 5′-TTCTAATACG ACTCACTATA AGGGAGGGCA GTGGGATGGC GTTAGTGAGG GAGGGTGTGG GGCGTCCAAC ACAT-3′. To generate truncated versions of FELIAP, nucleotides from both the 3′ and 5′ ends were sequentially removed as depicted schematically in Fig. 7A and B. Before use, all aptamer preparations were diluted into AF buffer, 10 mM Tris-Cl, 1 mM EDTA pH 8.0, Tris-buffered saline, or PPNE kinetics buffer (20 mM sodium phosphate, 100 mM NaCl, 0.1 mM EDTA, 0.1% polyethylene glycol (PEG) 8000, pH 7.4). The diluted aptamers were refolded by heating to 90 °C for 5 min and then cooled for 10 min on ice.

### Chromogenic Assay

Assays were performed in a 96-well flat bottom microtiter plate (Corning Incorporated, Corning, NY) at 37 °C in reaction buffer PPNE. Reactions (200 μL) contained 1 nM FXIa, 90 μM chromogenic substrate S2336 and aptamer concentrations ranging from 0.78 to 10 μM. The rate of substrate hydrolysis was recorded at 405 nm on an ELx808 Absorbance Microplate Reader (Biotek, Winooski, VT, USA).

### FXIa-Mediated FIX Activation

FIX (6.2 μM) was incubated with 2 nM FXIa in the presence of 10 μM FELIAP, 10 μM scrambled DNA or 2.6 μM KPI in TBS supplemented with 5 mM CaCl_2_ at 37 °C. Samples were incubated for 30 min. Following the 30 min incubation, the reactions were quenched using Sodium Dodecyl Sulphate (SDS) polyacrylamide gel electrophoresis (SDS-PAGE) loading buffer containing dithiothreitol and electrophoresed on a 12% SDS-polyacrylamide gel. Protein bands were visualized by staining with Coomassie Brilliant Blue.

### Inhibition of FXIa by antithrombin

FXIa (200 nM) was pre-incubated with 10 μM FELIAP or SCRAPT at 37 °C for 5 minutes, and then combined with 2 μM purified human antithrombin in the presence of 2 U/mL sodium heparin (Sigma-Aldrich) for a further 1 or 5 minutes. At the end of the antithrombin incubation time, samples were quenched and subjected to electrophoresis as described above.

### FXI activation assay

FXI (700 nM) in PPNE buffer was reacted with 70 nM thrombin in the presence or absence of 1 mg/L dextran sulphate (molecular weight 500 kDa) at 37 °C for 30 minutes in the presence or absence of 10 µM FELIAP or SCRAPT or 1.5 µM recombinant His-tagged hirudin variant 3 (HV3)^45^. At the conclusion of the reaction samples were quenched and subjected to electrophoresis as described above.

### Thrombin Generation Assay

Thrombin generation assays (TGA) were performed using either human normal pooled plasma (NPP) or FXI-depleted plasma (FXI-DP), (Haematologic Technologies, Essex Junction, VT, USA) with or without supplementation with FXIa. Three variations of TGA were performed, using: human normal pooled plasma (NPP) activated with silicates; FXI-DP activated with tissue factor; and FXI-DP supplemented with FXIa. All TGA reactions were performed in black flat bottom 96-well microtiter plates (Greiner Bio One). In the first protocol, 40 μL of NPP (diluted 1:5 in phosphate-buffered saline (PBS) was mixed with 10 μL of 300 μM FELIAP or SCRAPT) or PBS, 10 μL of APTT reagent (PTT-A, Diagnostica Stago, silica activator diluted 1:20 in PBS), 25 μL of 2 mM TGA substrate solution containing calcium chloride, and 15 μL of PBS, to comprise a total reaction volume of 100 μL. In the second protocol, the APTT reagent was substituted with the prothrombin time reagent Innovin (recombinant human tissue factor (Dade Behring, Deerfield, IL, USA) and NPP was substituted with FXI-DP. His-tagged recombinant hirudin variant 3 purified from *Pichia pastoris* ^45^ served as a positive control for TGA inhibition. In the third protocol, 1 µM FELIAP, SCRAPT, or KPI were combined with 0.25 nM FXIa (10 μL) and pre-incubated for 5 min at room temperature prior to combination with FXI-DP and activation with APTT reagent as in the first protocol. In all protocols, thrombin generation was then followed at 37 °C for 60 min at 1 min intervals using a Fluoroskan Ascent plate reader (Thermo Scientific) set to a wavelength of 380/460 nm. Data was imported into a Microsoft Excel evaluation spreadsheet (www.technoclone.com) for analysis and derivation of TGA test parameters.

### Modified APTT assay

APTT assays were performed using a STart 4 coagulometer (Diagnostica Stago) with some modifications. For the reactions, 5 μL of aptamer or PBS buffer control (FELIAP or SCRAPT, 30 μM) was heat-denatured and combined with an equal volume of varying concentrations of purified human FXIa at 37 °C for 3 min. APTT reagent (APTT-XL, 50 uL) was separately preincubated with 45 μL FXI-deficient human plasma under the same conditions, and then clotting was initiated by mixing both sets of components with 50 μL of 25 mM CaCl_2_ and the clotting time was determined.

### Surface Plasmon Resonance (SPR)

Kinetic measurements of aptamer binding were analyzed by SPR on a Biacore T200 (GE Healthcare). To immobilize the aptamer, a CM5 sensor chip (GE Healthcare) was activated with a 0.2 M *N-*ethyl-*N*′-(dimethylamino-propyl) carbodiimide (EDC) and 0.05 M *N-*hydroxysuccinimide (NHS) (Sigma) solution, followed by binding of streptavidin (0.2 mg/ml, pH 4.5). FELIAP and SCRAPT (3′ biotinylated) in 100 mM HEPES (pH 7.4), 150 mM NaCl, 0.01% Tween 20 were immobilized on the streptavidin-coated chips at a flow rate of 10 μL/min to 100 response units (RU). Flow cells were regenerated with 0.2% SDS (w/vol). FXIa at concentrations varying from 15.625 nM to 500 nM was injected at a flow rate of 50 μl/min for 180 s to monitor association, and HEPES buffer for 1800 s to monitor dissociation. The signal from the SCRAPT-immobilized flow cell was used as a reference and subtracted from the signal arising from FXIa binding to the FELIAP-immobilized cell. Binding of FXIa and aptamer was quantified by global analysis of on and off rates using the Langmuir 1:1 binding model, as determined with the instrument’s software provided by the manufacturer (Biacore). All experiments were done in triplicate.
